# Scientific evidence underlying the American College of Gastroenterology’s clinical practice guidelines

**DOI:** 10.1371/journal.pone.0204720

**Published:** 2018-10-03

**Authors:** Chase Meyer, Aaron Bowers, Cole Wayant, Jake Checketts, Jared Scott, Sanjeev Musuvathy, Matt Vassar

**Affiliations:** Oklahoma State University Center for Health Sciences, Tulsa, Oklahoma, United States of America; Flinders University, AUSTRALIA

## Abstract

**Background:**

Clinical practice guidelines contain recommendations for physicians to determine the most appropriate care for patients. These guidelines systematically combine scientific evidence and clinical judgment, culminating in recommendations intended to optimize patient care. The recommendations in CPGs are supported by evidence which varies in quality. We aim to survey the clinical practice guidelines created by the American College of Gastroenterology, report the level of evidence supporting their recommendations, and identify areas where evidence can be improved with additional research.

**Methods:**

We extracted 1328 recommendations from 39 clinical practice guidelines published by the American College of Gastroenterology. Several of the clinical practice guidelines used the differing classifications of evidence for their recommendations. To standardize our results, we devised a uniform system for evidence.

**Results:**

A total of 39 clinical practice guidelines were surveyed in our study. Together they account for 1328 recommendations. 693 (52.2%) of the recommendations were based on low evidence, indicating poor evidence or expert opinion. Among individual guidelines, 13/39 (33.3%) had no recommendations based on high evidence.

**Conclusion:**

Very few recommendations made by the American College of Gastroenterology are supported by high levels of evidence. More than half of all recommendations made by the American College of Gastroenterology are based on low-quality evidence or expert opinion.

## Introduction

It is estimated that 60 to 70 million Americans are affected by gastrointestinal (GI) diseases which may manifest as diarrhea, gas, bloating or abdominal pain.[1] These symptoms may be harmless, or they may be the result of more serious conditions like inflammatory bowel diseases. The American College of Gastroenterology (ACG) has developed clinical practice guidelines (CPGs) to help physicians diagnose and treat patients affected by diseases of the gastrointestinal (GI) tract. These CPGs contain recommendations for physicians to determine the most appropriate care for patients.[2] These guidelines systematically combine scientific evidence and clinical judgment, culminating in recommendations that have been shown to improve patient care.[3] The use of CPGs is far-reaching: they assist in making clinical decisions, furthering education, assessing quality of care, guiding resource allocation, and prioritizing research.[4]

Not all CPG recommendations should be given equal weight. The recommendations in CPGs contain supporting evidence which ranges from high quality (randomized controlled trials) to low quality (expert opinion). To grade the strength of recommendations and the quality of evidence underlying recommendations in guidelines, the ACG utilizes the Grading of Recommendations Assessment, Development and Evaluation (GRADE) approach.[5] By taking into account many factors such as: study limitations, inconsistency of results, indirectness of evidence, imprecision, and reporting bias between trials, the recommendation is given a grade (high, medium, low). This approach provides a universal and comprehensive system for rating quality of evidence that is increasingly being adopted worldwide and allows physicians and patients a means to quickly and confidently assess the quality behind recommendations.

With the increasing amount of CPGs, systematic reviews, and randomized controlled trials (RCTs), it is reasonable to assume that CPG recommendations would be based on a greater degree of high-quality evidence.[6] Unfortunately, this is often not true. Tricoci et al. reported that only 11% of the CPGs published by the American College of Cardiology (ACC) and American Heart Association (AHA) were based on high-grade evidence.[7] Only 30% of obstetrics and gynecology recommendations were based on high-level evidence.[8] The implication for these fields is clear: clinical decision making is often not based on findings from RCTs and systematic reviews. The ACG is devoted to the development of guidelines that are founded on principles of evidence-based medicine. There have been no studies surveying the quality of evidence in the guidelines developed by the ACG. Therein lies the rationale for our investigation: to examine the proportion of high, medium, and low-quality evidence to draw conclusions about the availability of evidence to gastroenterologists. The aim of our research was to review all of the guidelines affiliated with the ACG, report the level of evidence supporting their recommendations, and identify areas where evidence can be improved with additional research.

## Methods

### Identifying eligible guidelines

**Fig 1:**
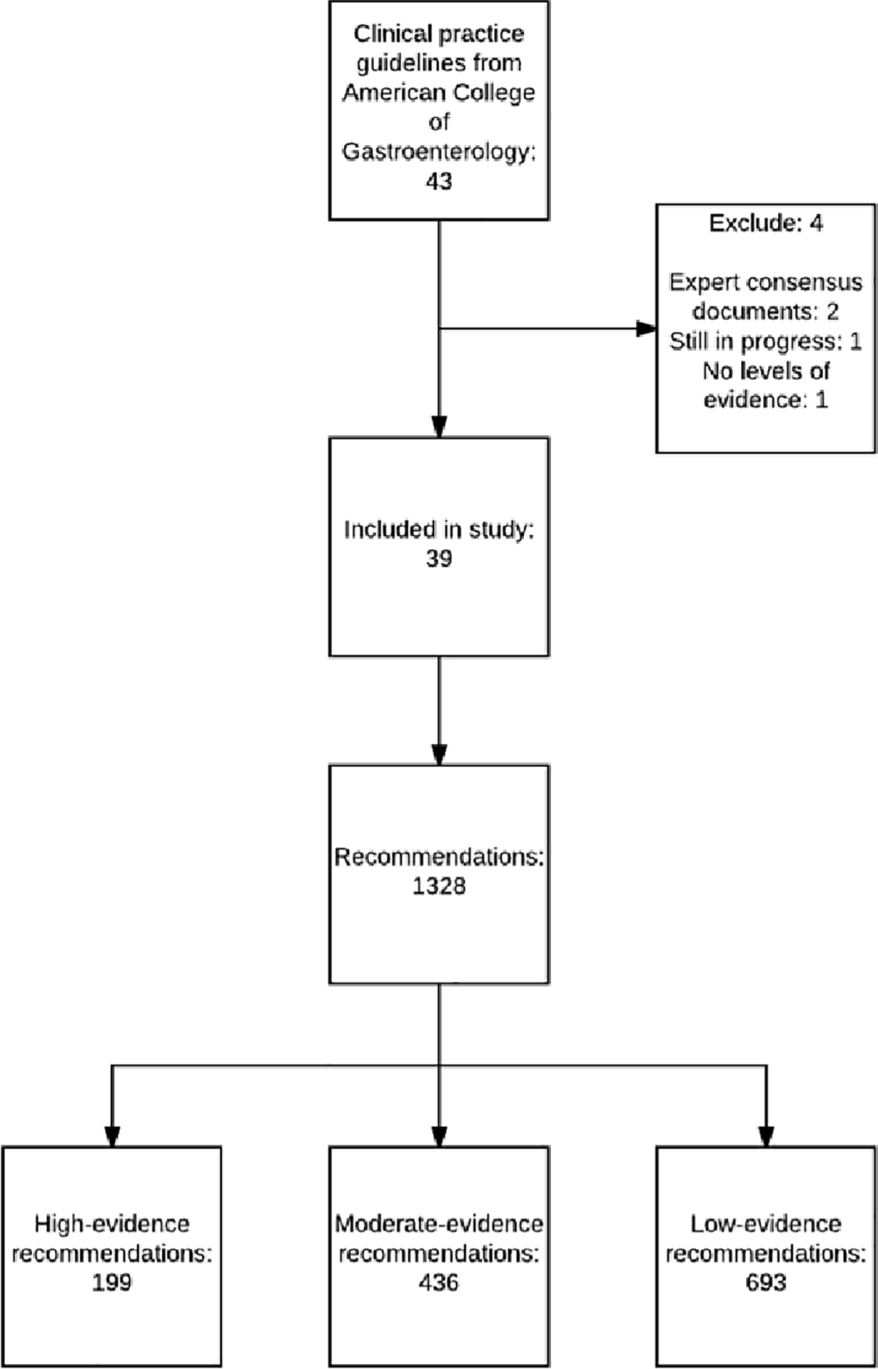
Flow diagram for inclusion and grading of recommendations.

The ACG has published 43 CPGs as of June 2017.[9] We obtained copies of the current CPGs from the ACG website in June 2017 (S1 Table).[10] In this study 39 CPG’s were used (Fig 1). Four were excluded due to being in progress (n = 1) or not providing levels of evidence for their recommendations (n = 3).

### Grading of recommendations

These CPGs provided a list of references and an assessment of individual study quality. Based on the results of these assessments, guideline authors assign a strength of evidence grade to each recommendation published in the summary of recommendations section in the CPG. The ACG uses the GRADE approach to assess strength of recommendations and the quality of evidence. While many of the classifications of evidence are uniform, there were several CPGs that used differing definitions (e.g., “High” in one guideline was defined as “further research is very unlikely to change our confidence in the estimate of effect” versus “data derived from multiple randomized clinical trials or meta-analyses” in another guideline). To standardize our results, we devised a uniform evidence taxonomy based on the varying definitions provided by authors of individual CPGs (Table 1 & S2 Table). Briefly, a grade of “High” was given if the evidence was based on randomized control trials without limitations, overwhelming evidence from observational studies, systematic reviews, meta-analyses, or if CPG authors concluded that further research was very unlikely to change their confidence in the estimate of effect. A grade of “Moderate” was given if evidence was based on one well-designed clinical trial, randomized control trials with limitations, cohort studies, case-control studies, or if authors believed that further research was likely to have an impact on the confidence in the estimate of effect. A grade of “Low” was given if the evidence was based on expert opinion, clinical experience, descriptive studies, case studies, poor-quality cohort studies, or if the authors expected further research to change the estimate of effect.

**Table 1:** The American College of Gastroenterology definitions for grading recommendations based off of the GRADE approach.

Definitions from ACG CPGs:	Corresponding grade:
Further research is very unlikely to change our confidence in the estimate of effect. Homogeneous evidence from multiple well-designed randomized (therapeutic) or cohort (descriptive) controlled trials, each involving a number of participants to be of sufficient statistical power. Data derived from multiple randomized clinical trials or meta-analyses. Evidence obtained from at least 1 well-designed and well-controlled randomized controlled trial that has either: a. Cancer end point with mortality or incidence, or b. Intermediate endpoint	High
Further research would be likely to have an impact on the confidence in the estimate of effect. Evidence from at least one large well-designed clinical trial with or without randomization, from cohort or case–control analytic studies, or well-designed meta-analysis. Data derived from a single randomized trial, or nonrandomized studies with limitations. Evidence obtained from well-designed and well-conducted cohort, case-control, or nonrandomized controlled trials that have: a. Cancer end point b. Intermediate end-point	Moderate
Further research would be expected to have an important impact on the confidence in the estimate of the effect and would be likely to change the estimate. Evidence based on clinical experience, descriptive studies, case series, or reports of expert committees. Recommendations are based on level 4 studies, meaning case series or poor-quality cohort studies. Any estimate of effect is very uncertain.	Low

### Data extraction and analysis

We conducted a content analysis based on the methodology of Wright et al.(8) SM obtained CPG’s produced by the ACG from their website. SM then extracted each recommendation into an Excel spreadsheet with its associated level of evidence. We also stratified the list by the guideline in which they were published along with the year of publication. Following extraction, we used the evidence taxonomy (Table 1) to assign the level of evidence as either high, moderate or low. All CPGs used high, moderate, and low whereas some CPGs used an additional grade of “very low.” We grouped “very low” into the “low” category to maintain three groups of grades throughout.” We then used the count function in Excel to find the number of recommendations supported by high, moderate and low level of evidence.

## Results

### Guideline characteristics

A total of 39 clinical practice guidelines were surveyed in our study. Together they account for 1328 recommendations. 693 (52.2%) of the recommendations were based on low evidence, indicating poor evidence or expert opinion. 436 recommendations (32.8%) were based on moderate evidence. 199 (15%) of recommendations were based on high evidence, such as those found in multiple randomized controlled trials or systematic reviews. Only one guideline—*Ulcerative Colitis in Adults[11]* - had over 50% of recommendations supported by high evidence.

### Strength of recommendations by year

**Fig 2:**
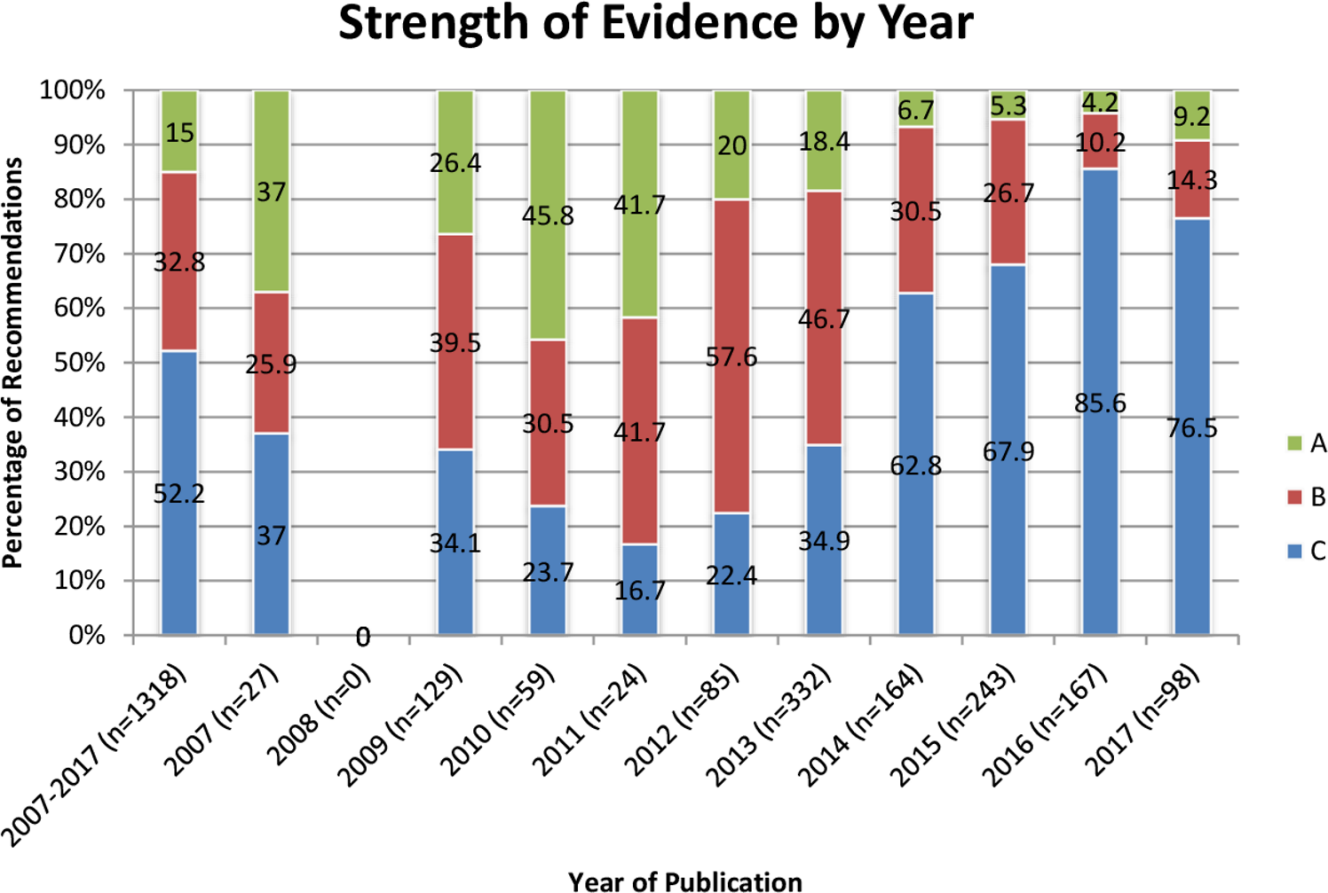
Strength of recommendation evidence stratified by year of guideline publication. Blue = High-grade evidence. Red = Moderate-grade evidence. Green = Low-grade evidence.

The year 2010 had the highest percentage of recommendations supported by high evidence (45.8%, 27/59), whereas 2016 had the lowest rate of high evidence (4.2%, 7/167). The year 2016 had the highest rate of low evidence (85.6%, 143/167), and 2011 had the fewest recommendations supported by low evidence (16.7%, 4/24). Between 2010 and 2016, the percentage of recommendations supported by high evidence decreased every year (45.8% to 4.2%). From 2011 to 2016, the rate of low evidence supporting published recommendations increased every year (16.7% to 85.6%). (Fig 2)

Among individual guidelines, 13 of 39 (33.3%) had no recommendations based on high evidence. These guidelines address the prevention, treatment, or management of colorectal cancer, GI bleeding, achalasia, liver disease, IBD, Lynch Syndrome, or others. Of these 13, four were based solely on low evidence.

## Discussion

The ACG has current guidelines for clinical practice dating back to 2007. The 39 guidelines we surveyed contained 1328 recommendations. Over half (52.2%) were based on low evidence, and only 15% were based on high evidence. A similar study conducted on the American College of Emergency Physicians (ACEP) CPGs found that less than 10% of their recommendations were based on high-quality evidence, and the majority of recommendations were based on expert opinion.[12] The American College of Chest Physicians (ACCP) found that only 0.4% of recommendations for treatment of thromboembolism were based on high-level evidence.[13] The amount of high-level evidence supporting the ACG guidelines compares favorably to the ACCP, ACEP, and ACC/AHA guidelines, but falls far short of the guidelines published by the American College of Obstetrics and Gynecology (ACOG). [7], [8]

RCTs are the cornerstone of clinical decision making, and the field of gastroenterology has a poor history of producing influential RCTs.[14] Nearly 25,000 randomized control trials are published each year, but given that 13 ACG guidelines have no high-evidence, it seems that few RCTs find their way into ACG clinical practice guidelines.[15] The disparity between the total number of gastroenterology RCTs and those that underpin guideline recommendations may be due to two factors: overlap between RCTs or practical barriers to conducting RCTs. The potential overlap between RCTs, otherwise known as research waste, may delay the advent of treatments for patients with preventable diseases.[16,17] For example, in our study we found that of the 13 guidelines with no high-quality evidence, all were focused on either prevention, treatment, or management. Each of these aspects of patient care may be tested in a randomized fashion. So, while some individual recommendations within these 13 guidelines may not be subjectable to an RCT due to ethical or practical concerns, at least one recommendation is.

For example, Koh et al. reported a 36% increase in recommendation number for the American Association for the Study of Liver Diseases (AASLD) since their development in 1998. But despite this substantial increase, less than 15% were based on high-grade evidence.[18]

Since 2003, the National Institutes of Health (NIH) budget for digestive disease research has plateaued, while corporate funding for gastroenterology research has dropped by more than 60% since 2008.[19,20] This translates into increased competition for grant applications, which are being awarded at the lowest rate in decades.[21] RCTs are among the most time-consuming and expensive research studies, but they produce the highest level of evidence. We suggest the ACG incentivize future research to strengthen recommendations that are currently supported by expert opinion or low-level evidence.

The goal of the ACG research program is to strengthen the capabilities of GI specialists, advance patient care, and create high-quality guidelines.[22] Some studies estimate that 54% to 70% of physicians consistently use CPGs in practice, so their quality is of high importance.[3,23] Therefore, a paucity of high-quality evidence affects physicians seeking evidence-based treatment options and patient seeking evidence-based care. The ACG has supported 612 investigators with over $18.8 million funded in research to date, but much of the funded research does not address the guideline recommendations with low evidence.[24] Recommendations that are based on low levels of evidence are important areas for research as they may expose patients to unnecessary risks and inflate health care costs.[25] Over $210 billion was spent on unnecessary health care services in 2009, representing 30% of wasted health care cost.[26] CPGs can give physicians a false sense of security, causing them to rely more on the guideline, than on critical-thinking and updated research.[15] This shows the importance of basing guideline recommendations on high-level evidence.

Low-level evidence recommendations are often based on expert opinion. When creating guidelines from expert consensus they are subject to bias. Conflicts of interest (COI) are potential sources of bias in the development of CPGs.[27] A 2017 study found that 55 of 101 (54.5%) authors writing CPGs in gastroenterology fields do not disclose their payments. Many of the recommendations in the ACG guidelines are based on low-level evidence that may be influenced by COI. We recommend that the ACG adopt a more stringent policy to address COI to minimize bias in clinical decision making.

Another major problem in basing recommendations on expert consensus is the fact that opinions vary between experts. This is illustrated in a study where Marras et al. found the highest percent of expert agreement on any recommendation was 81%.[28] Without further evidence validating one opinion over another, physicians will use their judgment to treat patients, leading to a variability in care. The number of ACG recommendations supported by low evidence and expert opinion highlights the need for further research leading to better evidence and improved patient outcomes.

## Limitations

Our study only evaluated CPGs published by the ACG and therefore is not generalizable outside of gastroenterology. Additionally, our study is not generalizable towards other ACG quality measures such as Appropriate Use Criteria, or other published literature. Because some of the guidelines were published before the current year, they may not be an accurate reflection of the current levels of evidence in gastroenterology literature, and therefore our study may underestimate the current research quality in the field. For some recommendations, establishing a randomized controlled trial may not be possible, and thus achieving a high level of evidence is unlikely.

## Conclusion

More than half of all recommendations made by the ACG are based on low-quality evidence or expert opinion. Thirteen of the 39 CPGs implemented by the ACG contain no recommendations supported by high-level evidence. Research should be focused on developing randomized control trials and systematic reviews to improve the evidence supporting CPGs.

## Supporting information

S1 Table Universalization of levels of evidence. (DOCX)

S2 Table A summary of the guidelines included in the analysis. (DOCX)
