# Supplementary material for: Scientific evidence underlying the American College of Gastroenterology’s clinical practice guidelines
Source: PLoS One. 2018 Oct 3;13(10):e0204720. doi: 10.1371/journal.pone.0204720 (PMC6169920; doi:10.1371/journal.pone.0204720)
Supplement: S1 Table — (DOCX) [file pone.0204720.s001.docx]

Supplementary table 1: Universalization of Levels of Evidence

| **Grade of Recommendation** | **Body of Evidence** | **Universal Grade** |
| --- | --- | --- |
| **Diagnosis and Management of Achalasia** | | |
| High: | Further research is very unlikely to change our confidence in the estimate of effect. | A |
| Moderate: | Further research is likely to have an important impact on our confidence in the estimate of effect and may change the estimate. | B |
| Low: | Further research is very likely to have an important impact on our confidence in the estimate of effect and is likely to change the estimate. | C |
| Very Low: | Any estimate of effect is very uncertain. | C |
| **Diagnosis, Treatment, and Prevention of Acute Diarrheal Infections in Adults** | | |
| High: | Further research is unlikely to change our confidence in the estimate of the effect. | A |
| Moderate: | Further research is likely to have an important impact and may change the estimate. | B |
| Low: | Further research is very likely to change the estimate. | C |
| Very Low: | Any estimate of effect is very uncertain. | C |
| **Management of Benign Anorectal Disorders** | | |
| High: | Further research is unlikely to alter our confidence about efficacy. | A |
| Moderate: | Further research is likely to affect future recommendations. | B |
| Low: | Further research is very likely to affect future assessments and recommendations. | C |
| **Diagnosis and Management of Barrett’s Esophagus** | | |
| High: | Further research was unlikely to change the authors’ confidence in the estimate of the effect. | A |
| Moderate: | Further research would be likely to have an impact on the confidence in the estimate of effect. | B |
| Low: | Further research would be expected to have an important impact on the confidence in the estimate of the effect and would be likely to change the estimate. | C |
| Very low: | Any estimate of effect is very uncertain. | C |
| **Diagnosis and Management of Small Bowel Bleeding** | | |
| High: | Further research was unlikely to change the authors’ confidence in the estimate of the effect. | A |
| Moderate: | Further research would be likely to have an impact on the confidence in the estimate of effect. | B |
| Low: | Further research would be expected to have an important impact on the confidence in the estimate of the effect and would be likely to change the estimate. | C |
| Very Low: | Any estimate of effect is very uncertain. | C |
| **Diagnosis and Management of Celiac Disease** | | |
| High: | Further research was unlikely to change the authors’ confidence in the estimate of the effect. | A |
| Moderate: | Further research would be likely to have an impact on the confidence in the estimate of effect. | B |
| Low: | Further research would be expected to have an important impact on the confidence in the estimate of the effect and would be likely to change the estimate. | C |
| Very Low: | Any estimate of effect is very uncertain. | C |
| **Diagnosis, Treatment, and Prevention of Clostridium difficile Infections** | | |
| High: | Further research is unlikely to change our confidence in the estimate of the effect. | A |
| Moderate: | Further research is likely to have an important impact and may change the estimate. | B |
| Low: | Further research is very likely to change the estimate. | C |
| **Epidemiology, Risk Factors, Patterns of Presentation, Diagnosis, and Management of Colon Ischemia (CI)** | | |
| High: | Further research was unlikely to change the authors’ confidence in the estimate of the effect. | A |
| Moderate: | Further research would be likely to have an impact on the confidence in the estimate of effect. | B |
| Low: | Further research would be expected to have an important impact on the confidence in the estimate of the effect and would be likely to change the estimate. | C |
| Very Low: | Any estimate of effect is very uncertain. | C |
| **Optimizing Adequacy of Bowel Cleansing for Colonoscopy: Recommendations From the US Multi-Society Task Force on Colorectal Cancer** | | |
| High: | Further research was unlikely to change the authors’ confidence in the estimate of the effect. | A |
| Moderate: | Further research would be likely to have an impact on the confidence in the estimate of effect. | B |
| Low: | Further research would be expected to have an important impact on the confidence in the estimate of the effect and would be likely to change the estimate. | C |
| Very low: | Any estimate of effect is very uncertain. | C |
| **Colonoscopy Surveillance after Colorectal Cancer Resection: Recommendations of the US Multi-Society Task Force on Colorectal Cancer 3** | | |
| High: | Further research was unlikely to change the authors’ confidence in the estimate of the effect. | A |
| Moderate: | Further research would be likely to have an impact on the confidence in the estimate of effect. | B |
| Low: | Further research would be expected to have an important impact on the confidence in the estimate of the effect and would be likely to change the estimate. | C |
| Very Low: | Any estimate of effect is very uncertain. | C |
| **Colorectal Cancer Screening**: Recommendations for Physicians and Patients from the U.S. Multi-Society Task Force on Colorectal Cancer | | |
| High: | Further research was unlikely to change the authors’ confidence in the estimate of the effect. | A |
| Moderate: | Further research would be likely to have an impact on the confidence in the estimate of effect. | B |
| Low: | Further research would be expected to have an important impact on the confidence in the estimate of the effect and would be likely to change the estimate. | C |
| Very Low: | Any estimate of effect is very uncertain. | C |
| **Management of Crohn’s Disease in Adults** | | |
| Grade A: | Homogeneous evidence from multiple well-designed randomized (therapeutic) or cohort (descriptive) controlled trials, each involving a number of participants to be of sufficient statistical power. | A |
| Grade B: | Evidence from at least one large well-designed clinical trial with or without randomization, from cohort or case–control analytic studies, or well-designed meta-analysis. | B |
| Grade C: | Evidence based on clinical experience, descriptive studies, or reports of expert committees. | C |
| **Management of Dyspepsia** | | |
| High: | Further research was unlikely to change the authors’ confidence in the estimate of the effect. | A |
| Moderate: | Further research would be likely to have an impact on the confidence in the estimate of effect. | B |
| Low: | Further research would be expected to have an important impact on the confidence in the estimate of the effect and would be likely to change the estimate. | C |
| Very Low: | Any estimate of effect is very uncertain. | C |
| **Guidelines for Colorectal Cancer Screening 2008** | | |
| High-quality evidence: | Randomized controlled trials without important limitations or overwhelming evidence from observational studies. | A |
| Moderate-quality evidence: | Randomized controlled trials with important limitations (inconsistent results, methodological flaws, indirect, or imprecise) or exceptionally strong evidence from observational studies. | B |
| Low quality or very low quality: | Observational studies or case series. | C |
| **Treatment of Helicobacter pylori Infection** | | |
| High: | Further research was unlikely to change the authors’ confidence in the estimate of the effect. | A |
| Moderate: | Further research would be likely to have an impact on the confidence in the estimate of effect. | B |
| Low: | Further research would be expected to have an important impact on the confidence in the estimate of the effect and would be likely to change the estimate. | C |
| Very Low: | Any estimate of effect is very uncertain. | C |
| **Diagnosis, Management, and Treatment of Hepatitis C: An Update** | | |
| Level A: | Data derived from multiple randomized clinical trials or meta-analyses. | A |
| Level B: | Data derived from a single randomized trial, or nonrandomized studies. | B |
| Level C: | Only consensus opinion of experts, case studies, or standard-of-care. | C |
| **An Update on Treatment of Genotype 1 Chronic Hepatitis C Virus Infection: 2011** | | |
| Level A: | Data derived from multiple randomized clinical trials or meta-analyses. | A |
| Level B: | Data derived from a single randomized trial, or nonrandomized studies. | B |
| Level C: | Only consensus opinion of experts, case studies, or standard-of-care. | C |
| **Genetic Testing and Management of Hereditary Gastrointestinal Cancer Syndromes** | | |
| High: | Further research was unlikely to change the authors’ confidence in the estimate of the effect. | A |
| Moderate: | Further research would be likely to have an impact on the confidence in the estimate of effect. | B |
| Low: | Further research would be expected to have an important impact on the confidence in the estimate of the effect and would be likely to change the estimate. | C |
| Very Low: | Any estimate of effect is very uncertain. | C |
| **Preventive Care in Inflammatory Bowel Disease** | | |
| High: | Further research was unlikely to change the authors’ confidence in the estimate of the effect. | A |
| Moderate: | Further research would be likely to have an impact on the confidence in the estimate of effect. | B |
| Low: | Further research would be expected to have an important impact on the confidence in the estimate of the effect and would be likely to change the estimate. | C |
| Very Low: | Any estimate of effect is very uncertain. | C |
| **Alcoholic Liver Disease** | | |
| Level A: | Data derived from multiple randomized clinical trials or meta-analyses. | A |
| Level B: | Data derived from a single randomized trial or nonrandomized studies. | B |
| Level C: | Only consensus opinion of experts, case studies, or standard of care. | C |
| **Diagnosis and Management of Non-alcoholic Fatty Liver Disease: Practice Guideline by the American Association for the Study of Liver Diseases, American College of Gastroenterology, and the American Gastroenterological Association 6** | | |
| High A: | Further research is unlikely to change confidence in the estimate of the clinical effect. | A |
| Moderate B: | Further research may change confidence in the estimate of the clinical effect. | B |
| Low C: | Further research is very likely to impact confidence on the estimate of clinical effect. | C |
| **The Diagnosis and Management of Idiosyncratic Drug-Induced Liver Injury** | | |
| High: | Further research was unlikely to change the authors’ confidence in the estimate of the effect. | A |
| Moderate: | Further research would be likely to have an impact on the confidence in the estimate of effect. | B |
| Low: | Further research would be expected to have an important impact on the confidence in the estimate of the effect and would be likely to change the estimate. | C |
| Very Low: | Any estimate of effect is very uncertain. | C |
| **Diagnosis and Management of Focal Liver Lesions** | | |
| High: | Further research was unlikely to change the authors’ confidence in the estimate of the effect. | A |
| Moderate: | Further research would be likely to have an impact on the confidence in the estimate of effect. | B |
| Low: | Further research would be expected to have an important impact on the confidence in the estimate of the effect and would be likely to change the estimate. | C |
| Very Low: | Any estimate of effect is very uncertain. | C |
| **Liver Disease and Pregnancy** | | |
| High: | Further research was unlikely to change the authors’ confidence in the estimate of the effect. | A |
| Moderate: | Further research would be likely to have an impact on the confidence in the estimate of effect. | B |
| Low: | Further research would be expected to have an important impact on the confidence in the estimate of the effect and would be likely to change the estimate. | C |
| Very Low: | Any estimate of effect is very uncertain. | C |
| **Guidelines on Genetic Evaluation and Management of Lynch Syndrome: A Consensus Statement by the US Multi-Society Task Force on Colorectal Cancer 2** | | |
| I: | Evidence obtained from at least 1 well-designed and well-controlled randomized controlled trial that has either:  a. Cancer end point with mortality or incidence, or  b. Intermediate end point | A |
| II: | Evidence obtained from well-designed and well-conducted nonrandomized controlled trials that have:  a. Cancer end point  b. Intermediate end point | B |
| III: | Evidence obtained from well-designed and well-conducted cohort or case-control studies with:  a. Cancer end point  b. Intermediate end point | B |
| IV: | Evidence from descriptive studies with:  a. Cancer end point  b. Intermediate end point | C |
| V: | Conclusions from authorities based on clinical experience, descriptive studies and/or expert committees. | C |
| **Evaluation of Abnormal Liver Chemistries** | | |
| High: | Further research was unlikely to change the authors’ confidence in the estimate of the effect. | A |
| Moderate: | Further research would be likely to have an impact on the confidence in the estimate of effect. | B |
| Low: | Further research would be expected to have an important impact on the confidence in the estimate of the effect and would be likely to change the estimate. | C |
| Very Low: | Any estimate of effect is very uncertain. | C |
| **Nutrition Therapy in the Adult Hospitalized Patient** | | |
| High: | We are very confident that the true effect lies close to that of the estimate of effect. | A |
| Moderate: | We are moderately confident in the effect estimate: the true effect is likely to be close to the estimate of effect, but there is a possibility that it is substantially different. | B |
| Low: | Our confidence in the effect estimate is limited: the true effect may be substantially different from the estimate of effect. | C |
| Very Low: | We have very little confidence in the effect estimate: the true effect is likely to be substantially different from the estimate of effect. | C |
| **Guidelines for Prevention of NSAID-Related Ulcer Complications** | | |
| A: | Strong evidence for multiple published, well-controlled randomized trials or a well-designed systematic meta-analysis. | A |
| B: | Strong evidence from at least one quality-published randomized controlled trial or evidence from published, well-designed, cohort or matched case – control studies. | B |
| C: | Consensus of authoritative expert opinions based on clinical evidence or from well designed, but uncontrolled or non-randomized clinical trials. | C |
| **Management of Acute Pancreatitis** | | |
| High: | Further research was unlikely to change the authors’ confidence in the estimate of the effect. | A |
| Moderate: | Further research would be likely to have an impact on the confidence in the estimate of effect. | B |
| Low: | Further research would be expected to have an important impact on the confidence in the estimate of the effect and would be likely to change the estimate. | C |
| Very Low: | Any estimate of effect is very uncertain. | C |
| **Primary Sclerosing Cholangitis** | | |
| High: | Further research was unlikely to change the authors’ confidence in the estimate of the effect. | A |
| Moderate: | Further research would be likely to have an impact on the confidence in the estimate of effect. | B |
| Low: | Further research would be expected to have an important impact on the confidence in the estimate of the effect and would be likely to change the estimate. | C |
| Very Low: | Any estimate of effect is very uncertain. | C |
| **Management of Patients With Ulcer Bleeding** | | |
| High: | Further research was unlikely to change the authors’ confidence in the estimate of the effect. | A |
| Moderate: | Further research would be likely to have an impact on the confidence in the estimate of effect. | B |
| Low: | Further research would be expected to have an important impact on the confidence in the estimate of the effect and would be likely to change the estimate. | C |
| Very Low: | Any estimate of effect is very uncertain. | C |
| **Ulcerative Colitis in Adults** | | |
| A: | Recommendations imply that there is consistent level 1 evidence (randomized controlled trials). | A |
| B: | Evidence would be level 2 or 3, which are cohort studies or case–control studies. | B |
| C: | Recommendations are based on level 4 studies, meaning case series or poor-quality cohort studies. | C |
| D: | Recommendations are based on level 5 evidence, meaning expert opinion. | C |
| **Prevention and Management of Gastroesophageal Varices and Variceal Hemorrhage in Cirrhosis** | | |
| A: | Data derived from multiple randomized clinical trials or meta-analyses. | A |
| B: | Data derived from a single randomized trial, or nonrandomized studies. | B |
| C: | Only consensus opinion of experts, case studies, or standard-of-care. | C |
| **Evidenced Based Approach to the Diagnosis and Management of Esophageal Eosinophilia and Eosinophilic Esophagitis (EoE)** | | |
| High: | Further research was unlikely to change the authors’ confidence in the estimate of the effect. | A |
| Moderate: | Further research would be likely to have an impact on the confidence in the estimate of effect. | B |
| Low: | Further research would be expected to have an important impact on the confidence in the estimate of the effect and would be likely to change the estimate. | C |
| Very Low: | Any estimate of effect is very uncertain. | C |
| **Role of Esophageal Stents in Benign and Malignant Diseases** | | |
| High: | Further research was unlikely to change the authors’ confidence in the estimate of the effect. | A |
| Moderate: | Further research would be likely to have an impact on the confidence in the estimate of effect. | B |
| Low: | Further research would be expected to have an important impact on the confidence in the estimate of the effect and would be likely to change the estimate. | C |
| Very Low: | Any estimate of effect is very uncertain. | C |
| **Diagnosis and Management of Gastroesophageal Reflux Disease** | | |
| High: | Further research was unlikely to change the authors’ confidence in the estimate of the effect. | A |
| Moderate: | Further research would be likely to have an impact on the confidence in the estimate of effect. | B |
| Low: | Further research would be expected to have an important impact on the confidence in the estimate of the effect and would be likely to change the estimate. | C |
| **Management of Patients With Acute Lower Gastrointestinal Bleeding** | | |
| High: | Further research was unlikely to change the authors’ confidence in the estimate of the effect. | A |
| Moderate: | Further research would be likely to have an impact on the confidence in the estimate of effect. | B |
| Low: | Further research would be expected to have an important impact on the confidence in the estimate of the effect and would be likely to change the estimate. | C |
| Very Low: | Any estimate of effect is very uncertain. | C |
| **Guidelines for Colonoscopy Surveillance After Screening and Polypectomy: A Consensus Update by the US Multi-Society Task Force on Colorectal Cancer** | | |
| High: | Further research was unlikely to change the authors’ confidence in the estimate of the effect. | A |
| Moderate: | Further research would be likely to have an impact on the confidence in the estimate of effect. | B |
| Low: | Further research would be expected to have an important impact on the confidence in the estimate of the effect and would be likely to change the estimate. | C |
| Very Low: | Any estimate of effect is very uncertain. | C |
