# Supplementary material for: Scientific evidence underlying the American College of Gastroenterology’s clinical practice guidelines
Source: PLoS One. 2018 Oct 3;13(10):e0204720. doi: 10.1371/journal.pone.0204720 (PMC6169920; doi:10.1371/journal.pone.0204720)
Supplement: S2 Table — (DOCX) [file pone.0204720.s002.docx]

Supplementary table 2: A Summary of the Guidelines included in the Analysis

| Guideline Title | Year of Adoption | Number of Recommendations | High | Moderate | Low |
| --- | --- | --- | --- | --- | --- |
| Diagnosis and Management of **Achalasia** | 2013 | 12 | 0 | 4 | 8 |
| Diagnosis, Treatment, and Prevention of **Acute Diarrheal Infections** in Adults | 2016 | 32 | 7 | 6 | 19 |
| Management of Benign **Anorectal** Disorders | 2014 | 40 | 4 | 23 | 13 |
| Diagnosis and Management of Barrett’s Esophagus | 2015 | 73 | 8 | 13 | 52 |
| Diagnosis and Management of Small **Bowel Bleeding** | 2015 | 52 | 3 | 13 | 36 |
| Diagnosis and Management of **Celiac Disease** | 2013 | 68 | 27 | 36 | 5 |
| Guidelines for Diagnosis, Treatment, and Prevention of **Clostridium difficile** Infections | 2013 | 80 | 16 | 33 | 31 |
| Epidemiology, Risk Factors, Patterns of Presentation, Diagnosis, and Management of **Colon Ischemia** (CI) | 2015 | 30 | 0 | 11 | 19 |
| Optimizing Adequacy of Bowel Cleansing for **Colonoscopy**: Recommendations From the US Multi-Society Task Force on Colorectal Cancer | 2014 | 30 | 7 | 7 | 16 |
| **Colonoscopy Surveillance after Colorectal Cancer Resection**: Recommendations of the US Multi-Society Task Force on Colorectal Cancer | 2016 | 5 | 0 | 1 | 4 |
| **Colorectal Cancer Screening** | 2009 | 15 | 0 | 11 | 4 |
| **Colorectal Cancer Screening**: Recommendations for Physicians and Patients from the U.S. Multi-Society Task Force on Colorectal Cancer | 2017 | 18 | 3 | 4 | 11 |
| Management of **Crohn’s Disease** in Adults | 2009 | 38 | 17 | 9 | 12 |
| Management of **Dyspepsia** | 2017 | 10 | 2 | 2 | 6 |
| Evidenced Based Approach to the Diagnosis and Management of Esophageal Eosinophilia and **Eosinophilic Esophagitis** (EoE) | 2013 | 21 | 1 | 7 | 13 |
| Role of **Esophageal Stents** in Benign and Malignant Disease | 2010 | 13 | 2 | 6 | 5 |
| Diagnosis and Management of **Gastroesophageal Reflux Disease** | 2013 | 63 | 6 | 30 | 27 |
| Management of Patients With Acute Lower **Gastrointestinal Bleeding** | 2016 | 27 | 0 | 0 | 27 |
| Management of **Gastroparesis** | 2013 | 34 | 7 | 16 | 11 |
| Treatment of **Helicobacter pylori** Infection | 2017 | 35 | 4 | 8 | 23 |
| Diagnosis, Management, and Treatment of **Hepatitis C**: An Update | 2009 | 70 | 15 | 29 | 26 |
| An Update on Treatment of Genotype 1 Chronic **Hepatitis C** Virus Infection: 2011 | 2013 | 24 | 10 | 10 | 4 |
| Genetic Testing and Management of **Hereditary Gastrointestinal Cancer Syndromes** | 2015 | 45 | 0 | 7 | 38 |
| Preventive Care in **Inflammatory** Bowel Disease | 2017 | 16 | 0 | 0 | 16 |
| Alcoholic **Liver** Disease | 2010 | 16 | 6 | 6 | 4 |
| Diagnosis and Management of Non-alcoholic Fatty **Liver** Disease: Practice Guideline by the American Association for the Study of **Liver** Diseases, American College of Gastroenterology, and the American Gastroenterological Association | 2012 | 45 | 6 | 34 | 5 |
| Diagnosis and Management of Idiosyncratic Drug-Induced **Liver** Injury | 2014 | 38 | 0 | 0 | 38 |
| Diagnosis and Management of Focal **Liver** Lesions | 2014 | 38 | 0 | 10 | 28 |
| **Liver** Disease and Pregnancy | 2016 | 36 | 0 | 4 | 32 |
| Evaluation of Abnormal **Liver** Chemistries | 2017 | 19 | 0 | 0 | 19 |
| Guidelines on Genetic Evaluation and Management of **Lynch Syndrome**: A Consensus Statement by the US Multi-Society Task Force on Colorectal Cancer | 2014 | 18 | 0 | 10 | 8 |
| Prevention of **NSAID-Related Ulcer** Complications | 2009 | 6 | 2 | 2 | 2 |
| **Nutrition** Therapy in the Adult Hospitalized Patient | 2016 | 67 | 0 | 6 | 61 |
| Management of Acute **Pancreatitis** | 2013 | 54 | 4 | 29 | 21 |
| **Primary Sclerosing Cholangitis** | 2015 | 42 | 2 | 20 | 20 |
| Management of Patients with **Ulcer Bleeding** | 2012 | 30 | 10 | 8 | 12 |
| **Ulcerative Colitis** in Adults | 2010 | 30 | 19 | 6 | 5 |
| Prevention and Management of Gastroesophageal **Varices and Variceal Hemorrhage in Cirrhosis** | 2007 | 27 | 10 | 7 | 10 |
| Guidelines for **Colonoscopy Surveillance** After Screening and Polypectomy: A Consensus Update by the US Multi-Society Task Force on Colorectal Cancer | 2012 | 10 | 1 | 7 | 2 |
